# Supplementary material for: Polyketide synthase gene clusters in the ericoid mycorrhizal fungus Oidiodendron maius: genome-wide prediction, expression profiling, and functional characterization of knockout mutants
Source: Front Plant Sci. 2026 Sep 16;17:1924430. doi: 10.3389/fpls.2026.1924430 (PMC13623903; doi:10.3389/fpls.2026.1924430)
Supplement: Supplementary file 1 [file SupplementaryFile1.zip › Supplementary Materials/Supplementary Folder 1.zip!/Oidma1_AssemblyScaffolds_Repeatmasked/knownclusterblast/cluster44/jgi.p_Oidma1_149276_mibig_hits.html]

| MIBiG Protein | Description | MIBiG Cluster | MiBiG Product | % ID | % Coverage | BLAST Score | E-value |
| --- | --- | --- | --- | --- | --- | --- | --- |
| ABC79591.2 | cercosporin\_toxin\_biosynthesis\_protein | BGC0001247\_c2 | Cercosporin\_biosynthetic\_gene\_cluster | 43 | 93.8073394495 | 328 | 1.3e-89 |
| CCT67993.1 | bikaverin\_cluster-O-methyltransferase | BGC0000030 | Bikaverin\_biosynthetic\_gene\_cluster | 37 | 97.247706422 | 314 | 2.6e-85 |
| CCH26291.1 | putative\_O-methyltransferase | BGC0001158\_c2 | Bikaverin\_biosynthetic\_gene\_cluster | 37 | 97.247706422 | 311 | 2.2e-84 |
| CCE67071.1 | methyltransferase | BGC0001242 | Fusarubin\_biosynthetic\_gene\_cluster | 42 | 86.9266055046 | 303 | 4.6e-82 |
| ADM79461.1 | O-methyltransferase | BGC0001266 | Grayanic\_acid\_biosynthetic\_gene\_cluster | 32 | 95.6422018349 | 201 | 1.9e-51 |
| ADM34151.1 | hypothetical\_protein | BGC0001084 | Notoamide\_/\_stephacidin\_biosynthetic\_gene\_cluster | 30 | 99.7706422018 | 181 | 2.6e-45 |
| AAS90052.1 | AflJ | BGC0000009 | Aflatoxin\_biosynthetic\_gene\_cluster | 30 | 101.834862385 | 174 | 2.5e-43 |
| AAS90004.1 | AflJ | BGC0000007 | Aflatoxin\_biosynthetic\_gene\_cluster | 30 | 91.7431192661 | 171 | 2.7e-42 |
| BAE71319.1 | possible\_transcriptional\_modulator | BGC0000004 | Aflatoxin\_biosynthetic\_gene\_cluster | 30 | 91.7431192661 | 170 | 4.7e-42 |
| AAS90027.1 | AflJ | BGC0000008 | Aflatoxin\_biosynthetic\_gene\_cluster | 30 | 91.7431192661 | 170 | 6.1e-42 |
| AAS90096.1 | AflJ | BGC0000006 | Aflatoxin\_biosynthetic\_gene\_cluster | 30 | 91.7431192661 | 170 | 6.1e-42 |
| AAS90072.1 | AflJ | BGC0000010 | Aflatoxin\_biosynthetic\_gene\_cluster | 30 | 91.7431192661 | 160 | 6.3e-39 |
| AAS90018.1 | OmtA | BGC0000007 | Aflatoxin\_biosynthetic\_gene\_cluster | 27 | 88.7614678899 | 119 | 7.2e-27 |
| AAS90065.1 | OmtA | BGC0000009 | Aflatoxin\_biosynthetic\_gene\_cluster | 28 | 79.8165137615 | 117 | 4.7e-26 |
| BAE71329.1 | o-methyltransferase\_A | BGC0000004 | Aflatoxin\_biosynthetic\_gene\_cluster | 27 | 88.9908256881 | 116 | 6.1e-26 |
| AAS90104.1 | OmtA | BGC0000006 | Aflatoxin\_biosynthetic\_gene\_cluster | 27 | 88.7614678899 | 115 | 1.8e-25 |
| AAS90041.1 | OmtA | BGC0000008 | Aflatoxin\_biosynthetic\_gene\_cluster | 27 | 88.9908256881 | 114 | 2.3e-25 |
| AAS90087.1 | OmtA | BGC0000010 | Aflatoxin\_biosynthetic\_gene\_cluster | 27 | 88.9908256881 | 114 | 4e-25 |
| ACH72896.1 | AflO | BGC0000011 | Aflatoxin/sterigmatocystin\_biosynthetic\_gene\_cluster | 27 | 72.7064220183 | 106 | 8.2e-23 |
| ADE22330.1 | O-methyltransferase | BGC0000065 | Galbonolides\_biosynthetic\_gene\_cluster | 26 | 88.3027522936 | 99 | 7.7e-21 |
| ADB02856.1 | AzicL | BGC0000202 | Azicemicin\_biosynthetic\_gene\_cluster | 28 | 69.0366972477 | 90 | 6.1e-18 |
| ABM21752.1 | PdmF | BGC0000256 | Pradimicin\_biosynthetic\_gene\_cluster | 28 | 44.7247706422 | 84 | 2.6e-16 |
| AAP69580.1 | putative\_O-methyltransferase | BGC0000226 | Gilvocarcin\_biosynthetic\_gene\_cluster | 24 | 38.9908256881 | 72 | 1.3e-12 |
| ADG86324.1 | O-methyltransferase | BGC0000190 | A-74528\_biosynthetic\_gene\_cluster | 27 | 49.3119266055 | 71 | 2.9e-12 |
| ABY83144.1 | Azi5 | BGC0000960 | Azinomycin\_B\_biosynthetic\_gene\_cluster | 30 | 33.2568807339 | 61 | 2.3e-09 |
